# 3β,6α-Diacet­oxy-5,9α-dihy­droxy-5α-cholest-7-en-11-one

**DOI:** 10.1107/S1600536813016206

**Published:** 2013-06-15

**Authors:** Vincenzo Piccialli, Angela Tuzi, Giorgia Oliviero, Nicola Borbone, Roberto Centore

**Affiliations:** a Dipartimento di Scienze Chimiche, Università di Napoli ’Federico II’, Complesso di Monte S. Angelo, Via Cinthia, 80126 Napoli, Italy; b Dipartimento di Farmacia, Università degli Studi di Napoli ’Federico II’, Via D. Montesano 49, 80131 Napoli, Italy

## Abstract

The title compound, C_31_H_48_O_7_, a polyoxygenated steroid, was obtained by chemical oxidation of 7-de­hydro­cholesteryl acetate. The mol­ecular geometry features *trans A*/*B* and *C*/*D* junctions at the steroid core with the acetyl groups in the equatorial position and a fully extended conformation for the alkyl side chain. A chair conformation is observed for rings *A* and *C* while ring *B* adopts a half-chair conformation. The five-membered ring *D* has an envelope conformation, with the C atom bearing the methyl group at the flap. The terminal isopropyl group and one acetyl group are disordered over two sets of sites with 0.774 (8):0.226 (8) and 0.843 (7):0.157 (7) ratios, respectively. An intra­molecular *S*(6) O—H⋯O hydrogen-bonding motif involving a hy­droxy donor and acceptor is observed. In the crystal, chains of mol­ecules running along the *b* axis are formed *via* O—H⋯O hydrogen bonds between hy­droxy donors and carbonyl acceptors of the ordered acetyl group, giving rise to a *C*(14) motif. The chains are wrapped around the 2_1_ screw axes.

## Related literature

For general information on the isolation of polyoxygenated steroids from marine source, see: Notaro *et al.* (1991 ▶, 1992 ▶). For the synthesis of polyoxygenated steroids, see: Migliuolo *et al.* (1992 ▶). For new selective oxidation protocols, see: Piccialli *et al.* (1993 ▶); Notaro *et al.* (1994 ▶); Caserta *et al.* (2005 ▶); Piccialli, D’Errico *et al.* (2013 ▶). For recent examples of hydrogen bonding in crystals, see: Centore, Fusco, Jazbinsek *et al.* (2013 ▶); Centore *et al.* (2013*a* ▶,*b* ▶); Centore, Fusco, Capobianco *et al.* (2013 ▶). For the structure and packing of the 6β isomeric steroid see: Piccialli, Oliviero *et al.* (2013 ▶).
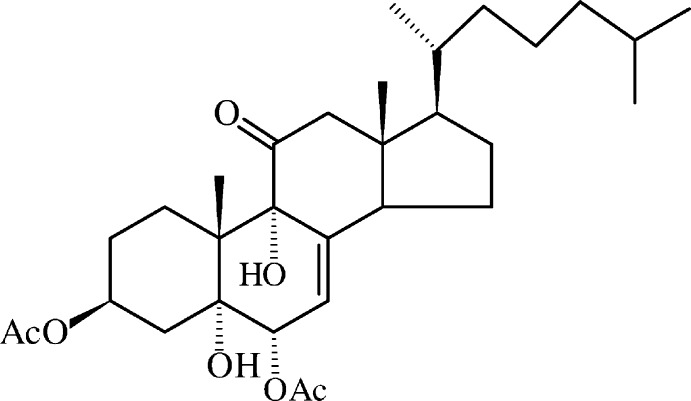


## Experimental

### 

#### Crystal data

C_31_H_48_O_7_
*M* *_r_* = 532.69
Monoclinic, 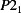

*a* = 10.964 (2) Å
*b* = 9.155 (1) Å
*c* = 14.740 (2) Å
β = 92.98 (1)°
*V* = 1477.5 (4) Å^3^
*Z* = 2
Mo *K*α radiation
μ = 0.08 mm^−1^
*T* = 173 K
0.60 × 0.35 × 0.05 mm

#### Data collection

Bruker–Nonius KappaCCD diffractometer
Absorption correction: multi-scan (*SADABS*; Bruker, 2001 ▶) *T*_min_ = 0.952, *T*_max_ = 0.996
9976 measured reflections
3498 independent reflections
2502 reflections with *I* > 2σ(*I*)
*R*_int_ = 0.051

#### Refinement

*R*[*F* ^2^ > 2σ(*F* ^2^)] = 0.047
*wR*(*F* ^2^) = 0.107
*S* = 1.07
3498 reflections
414 parameters
43 restraints
H atoms treated by a mixture of independent and constrained refinement
Δρ_max_ = 0.24 e Å^−3^
Δρ_min_ = −0.23 e Å^−3^

### 

Data collection: *COLLECT* (Nonius, 1999 ▶); cell refinement: *DIRAX/LSQ* (Duisenberg *et al.*, 2000 ▶); data reduction: *EVALCCD* (Duisenberg *et al.*, 2003 ▶); program(s) used to solve structure: *SIR97* (Altomare *et al.*, 1999 ▶); program(s) used to refine structure: *SHELXL97* (Sheldrick, 2008 ▶); molecular graphics: *ORTEP-3 for Windows* (Farrugia, 2012 ▶) and *Mercury* (Macrae *et al.*, 2006 ▶); software used to prepare material for publication: *WinGX* (Farrugia, 2012 ▶).

## Supplementary Material

Crystal structure: contains datablock(s) global, I. DOI: 10.1107/S1600536813016206/fj2632sup1.cif

Structure factors: contains datablock(s) I. DOI: 10.1107/S1600536813016206/fj2632Isup2.hkl

Additional supplementary materials:  crystallographic information; 3D view; checkCIF report

## Figures and Tables

**Table 1:** Hydrogen-bond geometry (Å, °)

*D*—H⋯*A*	*D*—H	H⋯*A*	*D*⋯*A*	*D*—H⋯*A*
O2—H1*D*⋯O1	0.84 (4)	1.89 (4)	2.619 (3)	145 (4)
O1—H1*C*⋯O7^i^	0.76 (4)	2.04 (4)	2.782 (3)	166 (4)

Symmetry code: (i) 
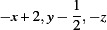
.

**Fig. 1.**
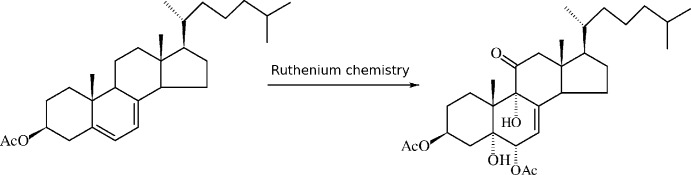
Synthesis of the title compound by ruthenium catalyzed oxidation of 7-dehydrocholesteryl acetate.

**Fig. 2.**
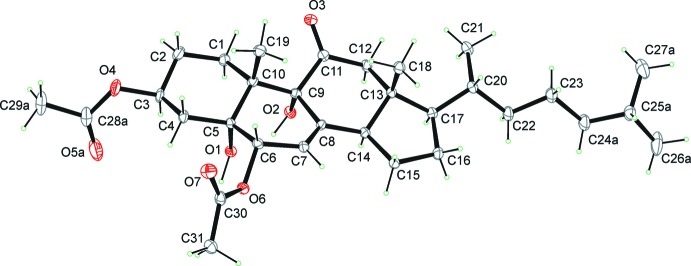
ORTEP view of the molecule, thermal ellipsoids are drawn at 30% probability level. The disordered parts with the minor occupancy factors were omitted.

**Fig. 3.**
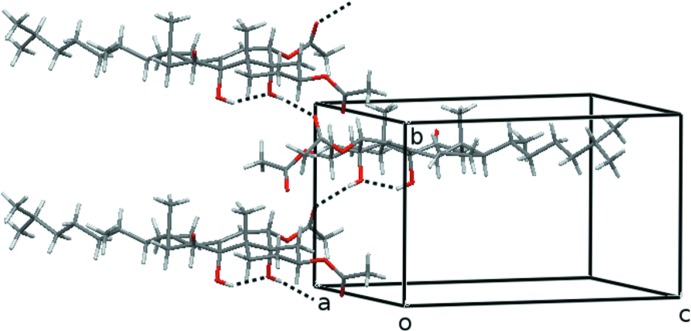
Partial packing showing chains of H bonded molecules running along b. The H bonds are represented by dashed lines. The disordered parts with the minor occupancy factors were omitted.

## supplementary crystallographic information

### Comment

Polyoxygenated steroids have been isolated from both marine and terrestrial sources. They are characterized by a wide range of oxygenation and nuclear substitution patterns. Some of them show antitumour activity as well as other important biological effects and, as a result, steroids are important targets from chemical, biological and medicinal point of view. Our group has previously been involved into studies aimed at the isolation of polyoxygenated steroids from marine sources (Notaro *et al.*, 1991, 1992), as well as at the synthesis and structural modification of some members of this class (Migliuolo *et al.*, 1992). This has led to the development of new efficient oxidation protocols, mostly based on the use of transition-metal oxo-species (Piccialli *et al.*, 1993; Notaro *et al.*, 1994; Caserta *et al.*, 2005; Piccialli, D'Errico *et al.*, 2013), to selectively introduce oxygenated functions into specific positions of the steroidal nucleus. Recently we have undertaken a study aimed at preparing new polyoxygenated steroids for structure-activity relationship studies. In this frame the title compound, shown in the Scheme, was synthesized from commercially available 7-dehydrocholesteryl acetate (Fig. 1) according to a previously developed RuO_4_-catalyzed route (Notaro *et al.*, 1994). Its 3β,5α,6α-oxygenation pattern is a motif found in some biologically active steroids isolated from sponges of genus *Dysidea* and, in particular, its C7—C10 functionalization pattern was seen as a key feature to introduce diversely configurated oxygenated functions at these carbon centres and/or neighbouring positions. The present X-ray diffraction study was undertaken in order to confirm the stereostructure of the title compound.

The molecular structure determined by X-ray analysis (Fig.2) fully confirms the stereostructure of the synthesized compound and shows an almost planar shape of the molecule. A chair conformation is observed both in A and C rings while the ring B, containing the C7=C8 double bond, adopts a half-chair conformation (twist at C5—C10 bond). The five-membered D ring has an envelope conformation, with C13 at the flap. In the steroid ring core *trans* junctions at A/B and C/D rings are observed. The two acetyl groups at C3 and C6 occupy equatorial positions of A and B rings. The alkyl side-chain is fully extended and the isopropyl group is disordered over two positions. Also in the acetyl moiety at C3 disorder in two positions is observed. An intramolecular H bonding motif S(6) involving hydroxy O2–H donor and hydroxy O1 acceptor is observed. In the crystal packing (Fig. 3) molecules are linked into chains running along *b*, through intermolecular H bonding between hydroxy O1–H donor and carbonyl O7 acceptor, giving rise to a C(14) motif. The chains are generated by the binary screw rotation of the space group. It is a remarkable finding that the isomeric 6β compound crystallizes with three independent molecules in the asymmetric unit (Piccialli, Oliviero *et al.*, 2013).

### Experimental

The title compound was prepared according to the recipe given in Notaro *et al.*, 1994. Crystals suitable for X-ray analysis were obtained by slow evaporation of CHCl_3_—MeOH (8:2) solutions of the compound.

### Refinement

H atoms of hydroxy groups were located in *DIF* maps and were refined with *U*_iso_ = 1.2×*U*_eq_ of the carrier atom. The positions of the other H atoms were determined stereochemically (C–H = 0.98–1.00 Å) and refined by the riding model with *U*_iso_=1.2×*U*_eq_ of the carrier atom (1.5 for H atoms of methyl group). Two different positions were found for the isopropyl group of the lateral alkyl chain (occupancy factor refined to 0.774 (8) for C25A/C26A/C27A; 0.226 (8) for C25B/C26B/C27B) and for the acetyl group at C3 (occupancy factor refined to 0.843 (7) for C28A/O5A/C29A; 0.157 (7) for C28B/O5B/C29B). SIMU and SAME restraints (Sheldrick, 2008) were applied to keep similar geometry in the disordered parts. Friedel pairs were merged using MERG3 and the absolute structure was assigned by reference to known chiral centers.

### Crystal data

**Table:**

C_31_H_48_O_7_	*F*(000) = 580
*M**_r_* = 532.69	*D*_x_ = 1.197 Mg m^−^^3^
Monoclinic, *P*2_1_	Mo *K*α radiation, λ = 0.71073 Å
Hall symbol: P 2yb	Cell parameters from 96 reflections
*a* = 10.964 (2) Å	θ = 3.7–18.4°
*b* = 9.155 (1) Å	µ = 0.08 mm^−^^1^
*c* = 14.740 (2) Å	*T* = 173 K
β = 92.98 (1)°	Block, colorless
*V* = 1477.5 (4) Å^3^	0.60 × 0.35 × 0.05 mm
*Z* = 2	

### Data collection

**Table:**

Bruker–Nonius KappaCCD diffractometer	3498 independent reflections
Radiation source: normal-focus sealed tube	2502 reflections with *I* > 2σ(*I*)
Graphite monochromator	*R*_int_ = 0.051
Detector resolution: 9 pixels mm^-1^	θ_max_ = 27.5°, θ_min_ = 3.2°
CCD rotation images, thick slices scans	*h* = −14→11
Absorption correction: multi-scan (*SADABS*; Bruker, 2001)	*k* = −11→10
*T*_min_ = 0.952, *T*_max_ = 0.996	*l* = −19→17
9976 measured reflections	

### Refinement

**Table:**

Refinement on *F*^2^	Primary atom site location: structure-invariant direct methods
Least-squares matrix: full	Secondary atom site location: difference Fourier map
*R*[*F*^2^ > 2σ(*F*^2^)] = 0.047	Hydrogen site location: inferred from neighbouring sites
*wR*(*F*^2^) = 0.107	H atoms treated by a mixture of independent and constrained refinement
*S* = 1.07	*w* = 1/[σ^2^(*F*_o_^2^) + (0.0293*P*)^2^ + 0.6674*P*] where *P* = (*F*_o_^2^ + 2*F*_c_^2^)/3
3498 reflections	(Δ/σ)_max_ = 0.004
414 parameters	Δρ_max_ = 0.24 e Å^−^^3^
43 restraints	Δρ_min_ = −0.23 e Å^−^^3^

### Special details

**Table:**

Geometry. All e.s.d.'s (except the e.s.d. in the dihedral angle between two l.s. planes) are estimated using the full covariance matrix. The cell e.s.d.'s are taken into account individually in the estimation of e.s.d.'s in distances, angles and torsion angles; correlations between e.s.d.'s in cell parameters are only used when they are defined by crystal symmetry. An approximate (isotropic) treatment of cell e.s.d.'s is used for estimating e.s.d.'s involving l.s. planes.
Refinement. Refinement of *F*^2^ against ALL reflections. The weighted *R*-factor *wR* and goodness of fit *S* are based on *F*^2^, conventional *R*-factors *R* are based on *F*, with *F* set to zero for negative *F*^2^. The threshold expression of *F*^2^ > σ(*F*^2^) is used only for calculating *R*-factors(gt) *etc*. and is not relevant to the choice of reflections for refinement. *R*-factors based on *F*^2^ are statistically about twice as large as those based on *F*, and *R*- factors based on ALL data will be even larger.

### Fractional atomic coordinates and isotropic or equivalent isotropic displacement parameters (Å^2^)

**Table:**

	*x*	*y*	*z*	*U*_iso_*/*U*_eq_	Occ. (<1)
C1	1.2313 (3)	0.6868 (7)	0.3113 (2)	0.0272 (8)	
H1A	1.2594	0.7237	0.3720	0.033*	
H1B	1.2185	0.5801	0.3166	0.033*	
C2	1.3314 (3)	0.7144 (7)	0.2447 (2)	0.0346 (9)	
H2A	1.3508	0.8200	0.2433	0.042*	
H2B	1.4065	0.6610	0.2650	0.042*	
C3	1.2894 (3)	0.6642 (7)	0.1518 (2)	0.0292 (8)	
H3	1.2725	0.5570	0.1533	0.035*	
C4	1.1750 (3)	0.7445 (7)	0.1178 (2)	0.0255 (7)	
H4A	1.1492	0.7092	0.0563	0.031*	
H4B	1.1923	0.8504	0.1139	0.031*	
C5	1.0716 (3)	0.7187 (6)	0.18291 (19)	0.0195 (7)	
C6	0.9551 (3)	0.8041 (6)	0.15326 (19)	0.0224 (7)	
H6	0.9748	0.9097	0.1444	0.027*	
C7	0.8587 (3)	0.7886 (6)	0.2195 (2)	0.0230 (7)	
H7	0.7773	0.8119	0.1994	0.028*	
C8	0.8780 (2)	0.7447 (6)	0.30446 (19)	0.0187 (6)	
C9	1.0055 (3)	0.7047 (6)	0.34476 (19)	0.0207 (7)	
C10	1.1079 (3)	0.7597 (6)	0.2832 (2)	0.0216 (7)	
C11	1.0205 (3)	0.7551 (6)	0.44532 (19)	0.0226 (7)	
C12	0.9153 (3)	0.7191 (6)	0.50437 (19)	0.0245 (7)	
H12A	0.9314	0.7613	0.5657	0.029*	
H12B	0.9085	0.6118	0.5108	0.029*	
C13	0.7943 (3)	0.7805 (6)	0.46246 (19)	0.0212 (7)	
C14	0.7768 (3)	0.7126 (6)	0.36678 (19)	0.0222 (7)	
H14	0.7775	0.6043	0.3760	0.027*	
C15	0.6462 (3)	0.7519 (7)	0.3353 (2)	0.0293 (8)	
H15A	0.6130	0.6816	0.2894	0.035*	
H15B	0.6419	0.8517	0.3095	0.035*	
C16	0.5767 (3)	0.7423 (7)	0.4239 (2)	0.0314 (8)	
H16A	0.5215	0.6567	0.4220	0.038*	
H16B	0.5272	0.8315	0.4316	0.038*	
C17	0.6749 (3)	0.7270 (7)	0.50405 (19)	0.0244 (7)	
H17A	0.6849	0.6204	0.5171	0.029*	
C18	0.8001 (3)	0.9459 (6)	0.4582 (2)	0.0256 (7)	
H18A	0.8630	0.9754	0.4171	0.038*	
H18B	0.8202	0.9849	0.5191	0.038*	
H18C	0.7207	0.9843	0.4357	0.038*	
C19	1.1215 (3)	0.9269 (6)	0.2916 (2)	0.0281 (8)	
H19A	1.1683	0.9507	0.3482	0.042*	
H19B	1.0404	0.9718	0.2923	0.042*	
H19C	1.1644	0.9644	0.2398	0.042*	
C20	0.6328 (3)	0.7998 (6)	0.5910 (2)	0.0246 (7)	
H20	0.6188	0.9058	0.5778	0.030*	
C21	0.7275 (3)	0.7879 (6)	0.6704 (2)	0.0294 (8)	
H21A	0.7452	0.6848	0.6830	0.044*	
H21B	0.6954	0.8336	0.7244	0.044*	
H21C	0.8026	0.8379	0.6548	0.044*	
C22	0.5109 (3)	0.7333 (7)	0.6181 (2)	0.0347 (8)	
H22A	0.4555	0.7270	0.5630	0.042*	
H22B	0.5266	0.6323	0.6397	0.042*	
C23	0.4456 (3)	0.8149 (7)	0.6905 (3)	0.0387 (9)	
H23A	0.5002	0.8212	0.7460	0.046*	
H23B	0.4287	0.9158	0.6691	0.046*	
C24A	0.3273 (3)	0.7443 (7)	0.7141 (3)	0.0438 (10)	0.774 (8)
H24A	0.2838	0.7146	0.6566	0.053*	0.774 (8)
H24B	0.3477	0.6538	0.7484	0.053*	0.774 (8)
C25A	0.2395 (4)	0.8302 (7)	0.7680 (3)	0.0349 (13)	0.774 (8)
H25B	0.2118	0.9134	0.7281	0.042*	0.774 (8)
C26A	0.1269 (6)	0.7425 (12)	0.7838 (8)	0.072 (3)	0.774 (8)
H26D	0.1489	0.6570	0.8211	0.108*	0.774 (8)
H26E	0.0895	0.7104	0.7253	0.108*	0.774 (8)
H26F	0.0686	0.8030	0.8153	0.108*	0.774 (8)
C27A	0.2935 (11)	0.8984 (13)	0.8535 (6)	0.068 (2)	0.774 (8)
H27D	0.2329	0.9626	0.8795	0.101*	0.774 (8)
H27E	0.3656	0.9557	0.8395	0.101*	0.774 (8)
H27F	0.3171	0.8216	0.8972	0.101*	0.774 (8)
C24B	0.3273 (3)	0.7443 (7)	0.7141 (3)	0.0438 (10)	0.226 (8)
H24C	0.3371	0.6379	0.7052	0.053*	0.226 (8)
H24D	0.2641	0.7775	0.6683	0.053*	0.226 (8)
C25B	0.2751 (15)	0.7655 (18)	0.8072 (12)	0.0349 (14)	0.226 (8)
H25A	0.3302	0.7091	0.8505	0.042*	0.226 (8)
C26B	0.281 (4)	0.921 (2)	0.840 (3)	0.068 (2)	0.226 (8)
H26A	0.3668	0.9473	0.8546	0.101*	0.226 (8)
H26B	0.2347	0.9304	0.8947	0.101*	0.226 (8)
H26C	0.2470	0.9856	0.7925	0.101*	0.226 (8)
C27B	0.1508 (17)	0.700 (4)	0.815 (3)	0.063 (10)	0.226 (8)
H27A	0.1318	0.6366	0.7623	0.095*	0.226 (8)
H27B	0.0897	0.7775	0.8167	0.095*	0.226 (8)
H27C	0.1496	0.6415	0.8707	0.095*	0.226 (8)
C30	0.9133 (3)	0.8178 (6)	−0.0083 (2)	0.0255 (7)	
C31	0.8490 (3)	0.7403 (7)	−0.0855 (2)	0.0333 (8)	
H31A	0.8661	0.7894	−0.1426	0.050*	
H31B	0.7608	0.7415	−0.0774	0.050*	
H31C	0.8776	0.6390	−0.0874	0.050*	
O1	1.0437 (2)	0.5655 (6)	0.18240 (15)	0.0226 (5)	
H1C	1.031 (3)	0.538 (4)	0.134 (2)	0.027*	
O2	1.0090 (2)	0.5488 (6)	0.35657 (15)	0.0234 (5)	
H1D	1.007 (3)	0.519 (4)	0.303 (3)	0.028*	
O3	1.1125 (2)	0.8138 (6)	0.47619 (15)	0.0310 (6)	
O4	1.3878 (2)	0.6926 (6)	0.09140 (16)	0.0394 (7)	
O5A	1.3419 (4)	0.4895 (7)	0.0110 (3)	0.0720 (16)	0.843 (7)
C28A	1.4072 (5)	0.5927 (7)	0.0263 (3)	0.0461 (18)	0.843 (7)
C29A	1.5189 (6)	0.6266 (12)	−0.0245 (5)	0.058 (2)	0.843 (7)
H29D	1.5884	0.5706	0.0017	0.086*	0.843 (7)
H29E	1.5369	0.7312	−0.0198	0.086*	0.843 (7)
H29F	1.5044	0.5999	−0.0885	0.086*	0.843 (7)
O5B	1.407 (2)	0.466 (2)	0.0706 (19)	0.0720 (17)	0.157 (7)
C28B	1.431 (3)	0.590 (2)	0.053 (3)	0.0461 (19)	0.157 (7)
C29B	1.538 (4)	0.636 (5)	−0.001 (4)	0.058 (3)	0.157 (7)
H29A	1.5166	0.7253	−0.0346	0.086*	0.157 (7)
H29B	1.5566	0.5579	−0.0433	0.086*	0.157 (7)
H29C	1.6088	0.6541	0.0407	0.086*	0.157 (7)
O6	0.90421 (18)	0.7413 (6)	0.06841 (13)	0.0239 (5)	
O7	0.9678 (2)	0.9317 (6)	−0.01271 (15)	0.0352 (6)	

### Atomic displacement parameters (Å^2^)

**Table:**

	*U*^11^	*U*^22^	*U*^33^	*U*^12^	*U*^13^	*U*^23^
C1	0.0224 (17)	0.038 (2)	0.0204 (15)	0.0016 (15)	−0.0016 (12)	0.0000 (15)
C2	0.0206 (17)	0.050 (2)	0.0335 (18)	−0.0059 (17)	0.0037 (14)	0.0041 (18)
C3	0.0181 (17)	0.044 (2)	0.0266 (17)	−0.0020 (15)	0.0118 (13)	0.0021 (16)
C4	0.0217 (16)	0.0338 (19)	0.0217 (15)	−0.0037 (15)	0.0080 (12)	0.0013 (16)
C5	0.0209 (16)	0.0218 (17)	0.0163 (14)	−0.0022 (13)	0.0044 (11)	−0.0005 (13)
C6	0.0252 (17)	0.0259 (18)	0.0161 (14)	−0.0028 (14)	0.0006 (12)	−0.0009 (14)
C7	0.0193 (16)	0.0287 (19)	0.0210 (15)	0.0019 (13)	0.0012 (12)	−0.0027 (14)
C8	0.0165 (15)	0.0199 (16)	0.0197 (15)	−0.0018 (13)	0.0027 (11)	−0.0002 (14)
C9	0.0232 (16)	0.0204 (18)	0.0181 (15)	0.0008 (13)	−0.0016 (12)	0.0006 (13)
C10	0.0187 (16)	0.0259 (18)	0.0202 (15)	−0.0029 (13)	0.0002 (12)	0.0024 (14)
C11	0.0214 (17)	0.0271 (18)	0.0187 (15)	0.0071 (14)	−0.0036 (12)	0.0014 (14)
C12	0.0254 (17)	0.033 (2)	0.0149 (14)	0.0031 (15)	0.0026 (12)	0.0014 (15)
C13	0.0207 (16)	0.0267 (19)	0.0164 (14)	−0.0005 (13)	0.0021 (11)	−0.0009 (13)
C14	0.0211 (16)	0.0301 (19)	0.0155 (14)	−0.0012 (14)	0.0020 (11)	−0.0008 (14)
C15	0.0198 (17)	0.049 (2)	0.0187 (15)	−0.0014 (16)	0.0012 (12)	−0.0057 (17)
C16	0.0223 (17)	0.051 (2)	0.0216 (16)	−0.0022 (17)	0.0040 (12)	−0.0052 (17)
C17	0.0240 (16)	0.0291 (19)	0.0205 (15)	−0.0021 (15)	0.0048 (12)	−0.0031 (15)
C18	0.0266 (18)	0.0278 (19)	0.0227 (16)	−0.0016 (14)	0.0051 (13)	0.0015 (15)
C19	0.0308 (19)	0.030 (2)	0.0242 (17)	−0.0075 (15)	0.0021 (14)	0.0003 (16)
C20	0.0274 (17)	0.0272 (18)	0.0196 (15)	0.0011 (14)	0.0046 (12)	−0.0008 (14)
C21	0.0363 (19)	0.031 (2)	0.0214 (16)	0.0057 (15)	0.0043 (13)	−0.0021 (15)
C22	0.0323 (19)	0.042 (2)	0.0313 (18)	−0.0052 (18)	0.0156 (14)	−0.0043 (18)
C23	0.035 (2)	0.040 (2)	0.043 (2)	−0.0067 (18)	0.0176 (16)	−0.0121 (19)
C24A	0.039 (2)	0.048 (2)	0.047 (2)	−0.009 (2)	0.0235 (17)	−0.010 (2)
C25A	0.033 (3)	0.036 (3)	0.037 (3)	−0.001 (2)	0.014 (2)	0.000 (2)
C26A	0.056 (5)	0.049 (6)	0.115 (10)	−0.007 (4)	0.058 (5)	−0.013 (6)
C27A	0.061 (4)	0.094 (5)	0.050 (4)	0.011 (4)	0.020 (3)	−0.023 (4)
C24B	0.039 (2)	0.048 (2)	0.047 (2)	−0.009 (2)	0.0235 (17)	−0.010 (2)
C25B	0.033 (3)	0.036 (3)	0.038 (3)	−0.001 (2)	0.014 (2)	0.000 (3)
C26B	0.061 (4)	0.094 (5)	0.050 (4)	0.011 (4)	0.020 (3)	−0.023 (4)
C27B	0.049 (15)	0.06 (2)	0.09 (2)	0.008 (13)	0.038 (13)	0.033 (15)
C30	0.0236 (17)	0.034 (2)	0.0197 (16)	0.0052 (16)	0.0040 (12)	0.0057 (15)
C31	0.0327 (19)	0.047 (2)	0.0201 (16)	−0.0001 (18)	0.0025 (13)	0.0009 (17)
O1	0.0261 (13)	0.0218 (12)	0.0201 (11)	−0.0021 (10)	0.0031 (9)	−0.0031 (10)
O2	0.0288 (13)	0.0225 (13)	0.0192 (11)	0.0017 (10)	0.0031 (9)	0.0009 (10)
O3	0.0267 (13)	0.0419 (15)	0.0239 (12)	−0.0032 (11)	−0.0029 (9)	−0.0074 (11)
O4	0.0250 (13)	0.0554 (18)	0.0391 (14)	−0.0021 (12)	0.0145 (10)	0.0044 (13)
O5A	0.084 (3)	0.067 (3)	0.069 (3)	−0.013 (2)	0.049 (3)	−0.022 (2)
C28A	0.038 (3)	0.059 (3)	0.044 (4)	0.010 (2)	0.021 (3)	0.006 (3)
C29A	0.045 (4)	0.079 (4)	0.052 (5)	0.019 (3)	0.031 (4)	0.023 (4)
O5B	0.084 (4)	0.067 (3)	0.069 (3)	−0.013 (3)	0.049 (3)	−0.022 (3)
C28B	0.038 (3)	0.059 (3)	0.044 (4)	0.010 (3)	0.021 (3)	0.006 (3)
C29B	0.045 (4)	0.079 (4)	0.052 (5)	0.019 (3)	0.031 (4)	0.023 (4)
O6	0.0253 (12)	0.0297 (13)	0.0167 (10)	−0.0024 (10)	0.0011 (8)	0.0017 (10)
O7	0.0441 (16)	0.0382 (16)	0.0232 (12)	−0.0102 (12)	0.0005 (10)	0.0098 (12)

### Geometric parameters (Å, º)

**Table:**

C1—C2	1.532 (4)	C20—C22	1.539 (4)
C1—C10	1.546 (4)	C20—H20	1.0000
C1—H1A	0.9900	C21—H21A	0.9800
C1—H1B	0.9900	C21—H21B	0.9800
C2—C3	1.494 (5)	C21—H21C	0.9800
C2—H2A	0.9900	C22—C23	1.514 (5)
C2—H2B	0.9900	C22—H22A	0.9900
C3—O4	1.458 (4)	C22—H22B	0.9900
C3—C4	1.516 (5)	C23—C24A	1.506 (5)
C3—H3	1.0000	C23—H23A	0.9900
C4—C5	1.540 (4)	C23—H23B	0.9900
C4—H4A	0.9900	C24A—C25A	1.501 (2)
C4—H4B	0.9900	C24A—H24A	0.9900
C5—O1	1.435 (4)	C24A—H24B	0.9900
C5—C6	1.542 (4)	C25A—C27A	1.500 (2)
C5—C10	1.557 (4)	C25A—C26A	1.501 (2)
C6—O6	1.461 (4)	C25A—H25B	1.0000
C6—C7	1.482 (4)	C26A—H26D	0.9800
C6—H6	1.0000	C26A—H26E	0.9800
C7—C8	1.322 (4)	C26A—H26F	0.9800
C7—H7	0.9500	C27A—H27D	0.9800
C8—C14	1.506 (4)	C27A—H27E	0.9800
C8—C9	1.534 (4)	C27A—H27F	0.9800
C9—O2	1.438 (4)	C25B—C27B	1.500 (2)
C9—C11	1.553 (4)	C25B—C26B	1.501 (2)
C9—C10	1.563 (4)	C25B—H25A	1.0000
C10—C19	1.542 (5)	C26B—H26A	0.9800
C11—O3	1.210 (4)	C26B—H26B	0.9800
C11—C12	1.517 (4)	C26B—H26C	0.9800
C12—C13	1.540 (4)	C27B—H27A	0.9800
C12—H12A	0.9900	C27B—H27B	0.9800
C12—H12B	0.9900	C27B—H27C	0.9800
C13—C18	1.517 (5)	C30—O7	1.205 (4)
C13—C14	1.544 (4)	C30—O6	1.339 (4)
C13—C17	1.553 (4)	C30—C31	1.487 (5)
C14—C15	1.525 (4)	C31—H31A	0.9800
C14—H14	1.0000	C31—H31B	0.9800
C15—C16	1.548 (4)	C31—H31C	0.9800
C15—H15A	0.9900	O1—H1C	0.76 (4)
C15—H15B	0.9900	O2—H1D	0.84 (4)
C16—C17	1.562 (4)	O4—C28B	1.20 (3)
C16—H16A	0.9900	O4—C28A	1.3500 (11)
C16—H16B	0.9900	O5A—C28A	1.2000 (10)
C17—C20	1.536 (4)	C28A—C29A	1.5003 (11)
C17—H17A	1.0000	C29A—H29D	0.9800
C18—H18A	0.9800	C29A—H29E	0.9800
C18—H18B	0.9800	C29A—H29F	0.9800
C18—H18C	0.9800	O5B—C28B	1.2000 (15)
C19—H19A	0.9800	C28B—C29B	1.5003 (15)
C19—H19B	0.9800	C29B—H29A	0.9800
C19—H19C	0.9800	C29B—H29B	0.9800
C20—C21	1.528 (4)	C29B—H29C	0.9800
			
C2—C1—C10	113.8 (3)	C16—C17—H17A	107.3
C2—C1—H1A	108.8	C13—C18—H18A	109.5
C10—C1—H1A	108.8	C13—C18—H18B	109.5
C2—C1—H1B	108.8	H18A—C18—H18B	109.5
C10—C1—H1B	108.8	C13—C18—H18C	109.5
H1A—C1—H1B	107.7	H18A—C18—H18C	109.5
C3—C2—C1	109.8 (3)	H18B—C18—H18C	109.5
C3—C2—H2A	109.7	C10—C19—H19A	109.5
C1—C2—H2A	109.7	C10—C19—H19B	109.5
C3—C2—H2B	109.7	H19A—C19—H19B	109.5
C1—C2—H2B	109.7	C10—C19—H19C	109.5
H2A—C2—H2B	108.2	H19A—C19—H19C	109.5
O4—C3—C2	107.6 (3)	H19B—C19—H19C	109.5
O4—C3—C4	109.7 (3)	C21—C20—C17	112.7 (3)
C2—C3—C4	111.4 (3)	C21—C20—C22	109.7 (3)
O4—C3—H3	109.4	C17—C20—C22	110.3 (3)
C2—C3—H3	109.4	C21—C20—H20	108.0
C4—C3—H3	109.4	C17—C20—H20	108.0
C3—C4—C5	110.0 (3)	C22—C20—H20	108.0
C3—C4—H4A	109.7	C20—C21—H21A	109.5
C5—C4—H4A	109.7	C20—C21—H21B	109.5
C3—C4—H4B	109.7	H21A—C21—H21B	109.5
C5—C4—H4B	109.7	C20—C21—H21C	109.5
H4A—C4—H4B	108.2	H21A—C21—H21C	109.5
O1—C5—C4	108.0 (2)	H21B—C21—H21C	109.5
O1—C5—C6	108.7 (2)	C23—C22—C20	115.9 (3)
C4—C5—C6	111.8 (3)	C23—C22—H22A	108.3
O1—C5—C10	106.5 (2)	C20—C22—H22A	108.3
C4—C5—C10	113.2 (2)	C23—C22—H22B	108.3
C6—C5—C10	108.3 (2)	C20—C22—H22B	108.3
O6—C6—C7	105.8 (2)	H22A—C22—H22B	107.4
O6—C6—C5	108.5 (2)	C24A—C23—C22	113.3 (3)
C7—C6—C5	111.9 (3)	C24A—C23—H23A	108.9
O6—C6—H6	110.2	C22—C23—H23A	108.9
C7—C6—H6	110.2	C24A—C23—H23B	108.9
C5—C6—H6	110.2	C22—C23—H23B	108.9
C8—C7—C6	124.6 (3)	H23A—C23—H23B	107.7
C8—C7—H7	117.7	C25A—C24A—C23	118.8 (3)
C6—C7—H7	117.7	C25A—C24A—H24A	107.6
C7—C8—C14	123.4 (3)	C23—C24A—H24A	107.6
C7—C8—C9	122.5 (3)	C25A—C24A—H24B	107.6
C14—C8—C9	113.7 (2)	C23—C24A—H24B	107.6
O2—C9—C8	107.6 (2)	H24A—C24A—H24B	107.1
O2—C9—C11	100.3 (2)	C27A—C25A—C26A	112.6 (4)
C8—C9—C11	110.4 (2)	C27A—C25A—C24A	115.2 (6)
O2—C9—C10	112.0 (2)	C26A—C25A—C24A	111.0 (5)
C8—C9—C10	111.4 (2)	C27A—C25A—H25B	105.7
C11—C9—C10	114.5 (2)	C26A—C25A—H25B	105.7
C19—C10—C1	109.1 (3)	C24A—C25A—H25B	105.7
C19—C10—C5	109.5 (3)	C27B—C25B—C26B	112.6 (5)
C1—C10—C5	109.1 (2)	C27B—C25B—H25A	105.4
C19—C10—C9	110.0 (3)	C26B—C25B—H25A	105.4
C1—C10—C9	110.5 (2)	C25B—C26B—H26A	109.5
C5—C10—C9	108.5 (2)	C25B—C26B—H26B	109.5
O3—C11—C12	121.6 (3)	H26A—C26B—H26B	109.5
O3—C11—C9	122.4 (3)	C25B—C26B—H26C	109.5
C12—C11—C9	115.9 (3)	H26A—C26B—H26C	109.5
C11—C12—C13	110.8 (2)	H26B—C26B—H26C	109.5
C11—C12—H12A	109.5	C25B—C27B—H27A	109.5
C13—C12—H12A	109.5	C25B—C27B—H27B	109.5
C11—C12—H12B	109.5	H27A—C27B—H27B	109.5
C13—C12—H12B	109.5	C25B—C27B—H27C	109.5
H12A—C12—H12B	108.1	H27A—C27B—H27C	109.5
C18—C13—C12	110.1 (3)	H27B—C27B—H27C	109.5
C18—C13—C14	111.6 (3)	O7—C30—O6	123.8 (3)
C12—C13—C14	106.4 (2)	O7—C30—C31	126.1 (3)
C18—C13—C17	111.7 (3)	O6—C30—C31	110.1 (3)
C12—C13—C17	116.9 (3)	C30—C31—H31A	109.5
C14—C13—C17	99.6 (2)	C30—C31—H31B	109.5
C8—C14—C15	118.6 (3)	H31A—C31—H31B	109.5
C8—C14—C13	114.7 (3)	C30—C31—H31C	109.5
C15—C14—C13	104.6 (2)	H31A—C31—H31C	109.5
C8—C14—H14	106.0	H31B—C31—H31C	109.5
C15—C14—H14	106.0	C5—O1—H1C	111 (3)
C13—C14—H14	106.0	C9—O2—H1D	102 (3)
C14—C15—C16	103.1 (2)	C28B—O4—C28A	20 (2)
C14—C15—H15A	111.2	C28B—O4—C3	118.2 (12)
C16—C15—H15A	111.2	C28A—O4—C3	117.5 (3)
C14—C15—H15B	111.2	O5A—C28A—O4	123.5 (4)
C16—C15—H15B	111.2	O5A—C28A—C29A	124.3 (4)
H15A—C15—H15B	109.1	O4—C28A—C29A	112.2 (5)
C15—C16—C17	107.0 (2)	O5B—C28B—O4	123 (2)
C15—C16—H16A	110.3	O5B—C28B—C29B	124.3 (4)
C17—C16—H16A	110.3	O4—C28B—C29B	112 (2)
C15—C16—H16B	110.3	C28B—C29B—H29A	109.5
C17—C16—H16B	110.3	C28B—C29B—H29B	109.5
H16A—C16—H16B	108.6	H29A—C29B—H29B	109.5
C20—C17—C13	119.2 (3)	C28B—C29B—H29C	109.5
C20—C17—C16	111.5 (3)	H29A—C29B—H29C	109.5
C13—C17—C16	103.6 (2)	H29B—C29B—H29C	109.5
C20—C17—H17A	107.3	C30—O6—C6	118.4 (3)
C13—C17—H17A	107.3		
			
C10—C1—C2—C3	−56.6 (4)	C9—C11—C12—C13	−55.5 (4)
C1—C2—C3—O4	−179.9 (3)	C11—C12—C13—C18	−63.1 (3)
C1—C2—C3—C4	59.8 (4)	C11—C12—C13—C14	58.0 (3)
O4—C3—C4—C5	−178.3 (3)	C11—C12—C13—C17	168.1 (3)
C2—C3—C4—C5	−59.3 (4)	C7—C8—C14—C15	−8.6 (5)
C3—C4—C5—O1	−63.0 (3)	C9—C8—C14—C15	178.2 (3)
C3—C4—C5—C6	177.4 (3)	C7—C8—C14—C13	−133.2 (3)
C3—C4—C5—C10	54.7 (4)	C9—C8—C14—C13	53.6 (4)
O1—C5—C6—O6	−51.2 (3)	C18—C13—C14—C8	61.1 (4)
C4—C5—C6—O6	68.0 (3)	C12—C13—C14—C8	−59.0 (4)
C10—C5—C6—O6	−166.6 (2)	C17—C13—C14—C8	179.1 (3)
O1—C5—C6—C7	65.2 (3)	C18—C13—C14—C15	−70.5 (3)
C4—C5—C6—C7	−175.6 (3)	C12—C13—C14—C15	169.4 (3)
C10—C5—C6—C7	−50.2 (3)	C17—C13—C14—C15	47.5 (3)
O6—C6—C7—C8	136.2 (3)	C8—C14—C15—C16	−165.5 (3)
C5—C6—C7—C8	18.2 (5)	C13—C14—C15—C16	−36.1 (4)
C6—C7—C8—C14	−172.3 (3)	C14—C15—C16—C17	10.4 (4)
C6—C7—C8—C9	0.3 (5)	C18—C13—C17—C20	−46.0 (4)
C7—C8—C9—O2	−108.6 (3)	C12—C13—C17—C20	82.1 (4)
C14—C8—C9—O2	64.7 (3)	C14—C13—C17—C20	−163.9 (3)
C7—C8—C9—C11	142.9 (3)	C18—C13—C17—C16	78.6 (3)
C14—C8—C9—C11	−43.8 (4)	C12—C13—C17—C16	−153.4 (3)
C7—C8—C9—C10	14.6 (4)	C14—C13—C17—C16	−39.3 (3)
C14—C8—C9—C10	−172.2 (3)	C15—C16—C17—C20	148.0 (3)
C2—C1—C10—C19	−68.7 (4)	C15—C16—C17—C13	18.5 (4)
C2—C1—C10—C5	50.9 (4)	C13—C17—C20—C21	−58.8 (4)
C2—C1—C10—C9	170.2 (3)	C16—C17—C20—C21	−179.4 (3)
O1—C5—C10—C19	−172.0 (2)	C13—C17—C20—C22	178.2 (3)
C4—C5—C10—C19	69.4 (3)	C16—C17—C20—C22	57.5 (4)
C6—C5—C10—C19	−55.2 (3)	C21—C20—C22—C23	67.4 (4)
O1—C5—C10—C1	68.6 (3)	C17—C20—C22—C23	−167.8 (3)
C4—C5—C10—C1	−50.0 (4)	C20—C22—C23—C24A	−179.9 (3)
C6—C5—C10—C1	−174.6 (3)	C22—C23—C24A—C25A	−165.9 (4)
O1—C5—C10—C9	−51.8 (3)	C23—C24A—C25A—C27A	−51.2 (7)
C4—C5—C10—C9	−170.5 (3)	C23—C24A—C25A—C26A	179.3 (6)
C6—C5—C10—C9	64.9 (3)	C2—C3—O4—C28B	120 (2)
O2—C9—C10—C19	−166.0 (3)	C4—C3—O4—C28B	−119 (2)
C8—C9—C10—C19	73.5 (3)	C2—C3—O4—C28A	142.1 (4)
C11—C9—C10—C19	−52.6 (3)	C4—C3—O4—C28A	−96.6 (4)
O2—C9—C10—C1	−45.4 (3)	C28B—O4—C28A—O5A	104 (4)
C8—C9—C10—C1	−166.0 (3)	C3—O4—C28A—O5A	6.5 (7)
C11—C9—C10—C1	67.9 (3)	C28B—O4—C28A—C29A	−77 (4)
O2—C9—C10—C5	74.2 (3)	C3—O4—C28A—C29A	−173.9 (4)
C8—C9—C10—C5	−46.4 (3)	C28A—O4—C28B—O5B	−102 (6)
C11—C9—C10—C5	−172.5 (3)	C3—O4—C28B—O5B	−9 (5)
O2—C9—C11—O3	109.9 (3)	C28A—O4—C28B—C29B	91 (5)
C8—C9—C11—O3	−136.8 (3)	C3—O4—C28B—C29B	−176 (3)
C10—C9—C11—O3	−10.2 (4)	O7—C30—O6—C6	4.9 (4)
O2—C9—C11—C12	−67.0 (3)	C31—C30—O6—C6	−176.1 (3)
C8—C9—C11—C12	46.3 (4)	C7—C6—O6—C30	131.8 (3)
C10—C9—C11—C12	173.0 (3)	C5—C6—O6—C30	−108.0 (3)
O3—C11—C12—C13	127.6 (3)		

### Hydrogen-bond geometry (Å, º)

**Table:**

*D*—H···*A*	*D*—H	H···*A*	*D*···*A*	*D*—H···*A*
O2—H1*D*···O1	0.84 (4)	1.89 (4)	2.619 (3)	145 (4)
O1—H1*C*···O7^i^	0.76 (4)	2.04 (4)	2.782 (3)	166 (4)

Symmetry code: (i) −*x*+2, *y*−1/2, −*z*.
